# Introduction of a Microsurgical In-Vivo Embolization-Model in Rats: The Aorta-Filter Model

**DOI:** 10.1371/journal.pone.0089947

**Published:** 2014-02-26

**Authors:** Lucas M. Ritschl, Andreas M. Fichter, Monika von Düring, David A. Mitchell, Klaus-Dietrich Wolff, Thomas Mücke

**Affiliations:** 1 Department of Oral and Maxillofacial Surgery, Technical University of Munich, Klinikum Rechts der Isar, Munich, Germany; 2 Department of Neuroanatomy, Ruhr University, Bochum, Germany; University of Louisville, United States of America

## Abstract

Vascular thrombosis with subsequent distal embolization remains a critical event for patients. Prevention of this life-threatening event can be achieved pharmacologically or mechanically with intravascular filter systems. The ability to evaluate the risk of embolization of certain techniques and procedures in vascular and microvascular surgery, such as, tissue glue or fibrin based haemostatic agents lacks convincing models. We performed 64 microvascular anastomoses in 44 rats, including 44 micro-pore polyurethane filter-anastomoses and 20 non-filter anastomoses. The rats were re-anesthetized and the aorta was re-exposed and removed four hours, three, seven, fourteen, thirty-one days, and six months postoperatively. The specimens were examined macro- and microscopically with regard to the appearance of the vessel wall, condition of the filter and the amount of thrombembolic material. Typical postoperative histopathological changes in vessel architecture were observed. Media necrosis was the first significant change three days postoperatively. Localized intimal hyperplasia, media necrosis, increase of media fibromyocytes and adventitial hypercellularity were seen to a significant extent at day seven postoperatively. Significant neovascularization of adventitia adjacent to the filter was seen after 14 days. A significant amount of thrombotic material was seen after four hours, three and 14 days interval. Only three intravascular filters became completely occluded (6.82%). The *aorta-filter-anastomosis model* appeared to be a valid in-vivo model in situations at risk for thrombembolic events, for microsurgical research and allowed sensitive analysis of surgical procedures and protection of the vascularized tissue. It may be suitable for a wide range of in-vivo microvascular experiments particularly in the rat model.

## Introduction

Distal embolization of plaque or thrombotic material may occur spontaneously during the complex process of plaque formation and rupture, followed by subsequent infarction or ischemic complications [1]. Surgical interventions in atherosclerotic vessels create a high risk for thromboembolic complications and their consequences [2], [3]. The detection of these events has been made possible by different imaging technologies, such as magnetic resonance imaging, computed tomography, myocardial contrast echography and transcranial Doppler [4], . The linkage between (micro-) vascular obstruction and unfavourable long-term outcomes has been evaluated for multiple vascular and microvascular sites [7], [8], [9], [10], [11]. In general, thrombogenesis and distal embolization can be prevented by pharmacological or mechanical approaches [12], [13]. Complex surgery particularly microvascular reconstruction in confined areas where platelet inhibition and/or anticoagulation may create a risk for bleeding and haematoma formation which compromises the procedure traditionally avoid the pharmacological route although this is routine in general vascular surgery [14]. Protection from emboli by mechanical filters can be applied surgically or endovascularly [15], [16]. The concept of mechanical emboli protection is based on a filter system between the potential embolic source and the distal vascular bed by filtration of the flowing blood.

Filter-based embolic protection devices are used with a pore size of 100–150 µm. These devices can be applied within vessels of about 3–5 mm in diameter [16], [17]. During microvascular anastomoses direct application of a filter in the vessel may also be used for the prevention or detection of microvascular thromboembolic complications following vascular procedures.

The purpose of this study was to establish a reliable and reproducible microvascular *in-vivo model* that enables avoidance of arterial embolization as well as further investigation of the embolic risk of different microsurgical techniques and procedures.

## Methods and Materials

### Ethical Statement

The study was conducted in conformance with current German regulations and guidelines for animal welfare and to the international principles of laboratory animal care. The animal experiments were approved by the “Regierung von Oberbayern” (Az. 55.2-1-54-2531-93-10).

Animals were housed in filter-top cages under SPF-conditions according to the FELASA guidelines at 22±1°C, 46±20% relative humidity and a 12 hours light/dark cycle in the center for preclinical research of the Technical University of Munich. Water and standard rodent diet (Altromin; Altromin Spezialfutter GmbH & Co. KG; Lage, Germany) was given ad libitum. Animals were allowed to acclimatize to their surrounding for at least seven days before procedures were started. The rats were visited twice a day by one of the team (LMR, AMF or TM) postoperatively and a veterinarian observing the animals routinely. The daily observations were carried out according to a standardized protocol regarding animal welfare (pain reaction, animal’s gesture and behaviour), wound healing of the abdomen and especially the movement of the hind legs.

All animals were sacrificed with intracardial injection of pentobarbital 60 mg/kg body weight (Narcoren®, Fa. Rhone. Merieux GmbH, Laupheim) and exsanguinations following the defined observation period according to Close et al. [18].

### Operation

**Figure 1:**
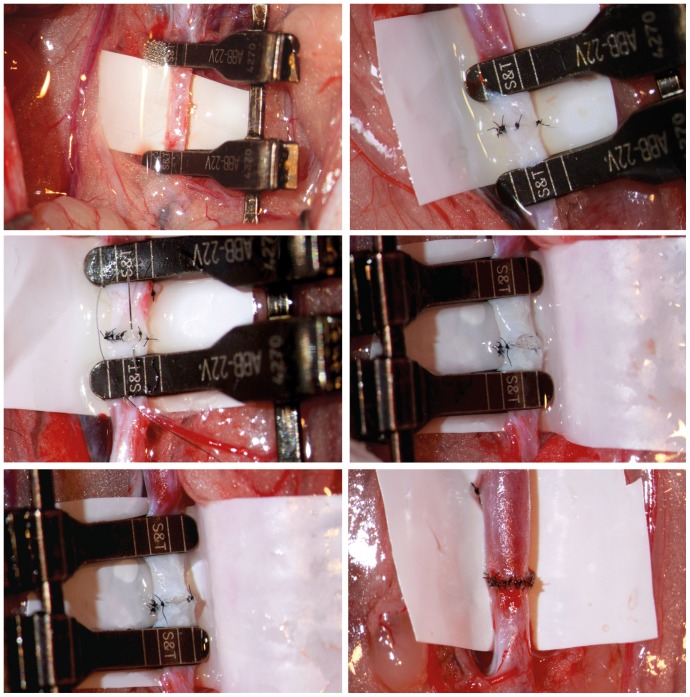
Microvascular anastomosis with introduction of the micro-pore polyurethane filter: *upper left picture* un-dissected aorta with the filter on the proximal clamp, *upper right and middle left pictures* anastomosis of the front wall, *middle right and lower left pictures* anastomosis of the back wall with visible intravascular filter, *and lower right picture* completed aortal-filter anastomosis. *(16x magnification)*.

The operations were performed by two experienced microsurgeons (LMR and TM) on male Wistar rats weighing between 300 and 350 g. The Wistar rats were anesthetized with intraperitoneal injection of ketamine 10% (1 mL/kg/weight) and xylazine 2% (0.25 mL/kg/weight) supplemented by quarter dose when needed [19]. The infrarenal aorta was exposed and freed from surrounding tissues. Microanastomosis and insertion of the micro-pore polyurethane filter (Joseph Schimmel GmbH & Co; Aldesheim, Germany) was carried out 3–5 mm proximal to aortic bifurcation with 10-0 Ethilon (Ethicon, Johnson & Johnson; Livingston, Scotland) interrupted sutures (**Figure 1** **)**. The diameter of the polyurethane filter micro-pores was 100 µm and the filter itself had no special surface coating. Blood loss following anastomosis was aspirated in a syringe with an atraumatic needle and documented.

Two groups were created by random number generation and the rats allocated to Group 1 or Group 2.

#### Group I

The abdomen was closed and the rats were observed for a postoperative interval. The vessel including the filter anastomosis was then re-exposed, removed and prepared for histologic section three, seven and 31 days and six months, according to the subgroup. Each subgroup of group I consisted of six rats.

#### Group II

**Figure 2:**
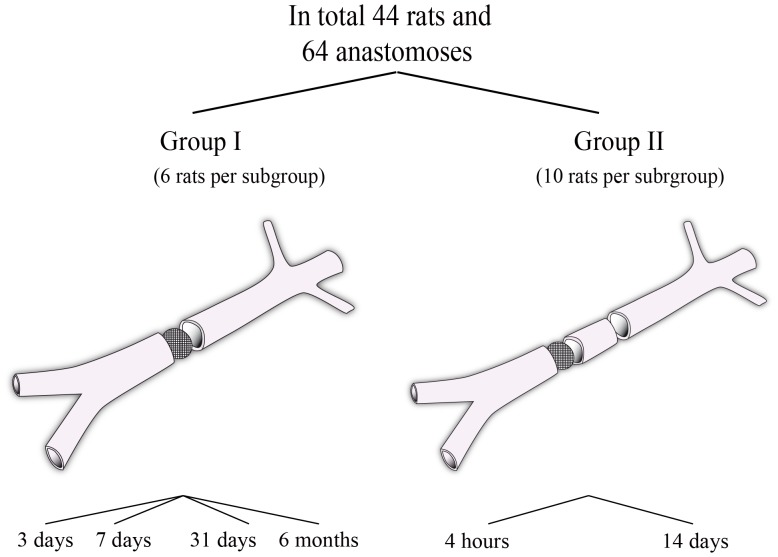
Overview of the groups I and II. Group I evaluated the healing process of the filter anastomosis alone. Group II evaluated the possibility to perform two adjacent anastomoses.

An additional microanastomosis was performed proximal to the filter anastomosis using 10-0 Ethilon for interrupted sutures followed by closure of the abdomen. The vessels including the filter and proximal non-filter anastomosis were re-exposed, removed and prepared for histological examination at the following postoperative intervals: four hours and 14 days (**Figure 2** **)**. Each subgroup in group II consisted of ten rats. Two anastomoses were performed in each infrarenal aorta (n = 40). The rats did not receive anticoagulation at any time in the pre-. intra- or postoperative phases.

### Postoperative Analysis

Macroscopic thrombembolic clots were measured within the filter and isovolumetrically evaluated (Image Processing and Analysis in Java; Image J 1.41; National Institutes of Health; Bethesda, Maryland, USA).

The entire specimen was then fixed with formaldehyde 4% in 100 mM phosphate buffer solution before embedding in araldite. Subsequently the specimens were fixed in 2.5% glutaraldehyde and dehydrated in a graded ethanol series and critical point dried in osmium tetroxide. After embedding in araldite the specimens were cut with a Microtom UltracCut E (series Reichert-Jung, Leica, Vienna, Austria) into 0.75 µm thick sections. A total number of 10–40 sections of each specimen was obtained and subsequently stained with toluidine blue (Fluka, Buchs, Switzerland) [20].

A blinded specialist histoanatomist conducted the postoperative histological analysis with the help of a structured protocol for defined histologic changes in accordance to a previously published protocol by Chow et al. [21] (**Table 1** **)**. The area 500 µm between the proximal and distal part of the anastomosis were analyzed specifically. The results were documented with a digital camera (CAMEDIA C5050; Olympus, Hamburg, Germany).

**Table 1:** Criteria for the histological assessment of vascular specimen judged by two independent observers. The different vessel layers were scored as described.

Vessel layer	Histologic criteria (score range)	Assessment
**Intima**	Intimal hyperplasia (0–4)	**0 = **≤ 2 layers and no expansion, **1** = >2 and ≤ 5 layers and expansion of ≤ 300 µm in length,**2** = >5 layers and expansion of >300 µm in length
	Interruption of internal elastic lamina(0–2)	**0** = none, **1** = <200 µm, **2** = >200 µm
**Media**	Cellular infiltration (0–2)	**0** = ≤ 1 cell, **1** = >1 and ≤ 5 cells, **2** = >5 cells
	Necrosis (0–2)	**0** = none, **1** = beginning, **2** = complete
**Adventitia**	Cellular infiltration (0–2)	**0** = ≤ 1 cell, **1** = >1 and ≤ 5 cells, **2** = >5 cells
	Neovascularization and lymphangiogenesis(0–2)	**0** = ≤ 1 vessel, **1** = >1 and ≤ 3 vessels, **2** = >3 vessels
**Filter**	Patent	not (0), reduced (1), yes (2)

### Statistical Analyses

Criteria for evaluation statistically are presented in **Table 1**. The Man-Whitney-U-Test was used for statistical analysis of histological findings. The different subgroups were compared to each other to have a statistically validated wound healing process and to make the results comparable to other studies in the literature.

The amount of thrombembolic material was statistically analyzed using the t-test.

All variables are expressed as mean ± standard deviation (SD). Data were analyzed with Statistical Product and Service Solutions (SPSS for Windows, release 18.0.0, 2010, SPSS Inc.; Chicago, USA) and Microsoft ® Office Excel (Microsoft Excel for Windows, release 11., 2003, Microsoft Corporation).

## Results

In total, 64 anastomoses were performed in 44 rats. Three filters were found to be occluded (6.82%), one in each of the four hours, 14 and 31 days sub-group. 25.42±1.14 interrupted sutures were needed to accomplish the filter-anastomosis in 51.78 minutes ±8.08 in group I. The filter anastomoses in group II were performed in 54.12 minutes ±11.04 in group II, respectively. The proximal non-filter anastomoses in group II were achieved with 16.2±2.09 interrupted sutures in 37.34 minutes ±14.15, in average. No significant difference in total blood loss was seen (0.68 ml ±0.36 in group I vs. 0.64 ml ±0.33 in group II), **Table 2**.

**Table 2:** Operative results of the groups I and II.

Group	Mean time for anastomosisin min (SD)	Mean interruptedsutures (SD)	Mean total blood lossin ml (SD)	Patency
**Group I 3 days**	48.97 (7.57)	25.3 (1.03)	0.72 (0.21)	6/6
**Group I 7 days**	55.62 (10.28)	26 (0.89)	0.92 (0.58)	6/6
**Group I 31 days**	48.95 (8.2)	24.83 (1.17)	0.44 (0.22)	5/6
**Group I 6 months**	57.15 (1.62)	25.5 (1.38)	0.65 (0.14)	6/6
**Average**	51.78 (8.08)	25.42 (1.14)	0.68 (0.36)	23/24 (95,83%)
**Group II**	**Mean time for anastomosis** **proximal/distal in min (SD)**	**Mean interrupted sutures** **proximal/distal (SD**	**Mean total blood loss** **in ml (SD)**	**Patency**
**Group II 4 hours**	43.22 (17.4)/58.51 (12.64)	15 (1.7)/24.5 (1.27)	0.56 (0.28)	9/10 (90%)
**Group II 14 days**	31.47(6.57)/49.75 (7.4)	17.4 (1.78)/24.3 (0.82)	0.73 (0.38)	9/10 (90%)
**Average**	37.34 (14.15)/54.13 (11.04)	16.2 (2.09)/24.4 (1.05)	0.64 (0.33)	18/20 (90%)

### Group I

23 out of 24 vessels were patent (95,83%). Thrombo-embolic clots in the filter device was only found in sub-groups three and seven days postoperatively and showed a significant difference compared to the 14, 31 days and six months sub-groups (each p = 0.031) (**Table 3** **)**.

**Table 3:** Statistical analysis of the histological changes compared to the four hours sub-group using the Mann-Whitney-U Test and T-Test for T*.

Group	IH	IIEL	MFI	N	AH	NL	ATEM [mm^3^]	T*
**I-3 days**	1.000	0.713	1.000	0.000	1.000	1.000	0.03971	0.331
**I-7 days**	0.005	0.713	0.031	0.000	0.000	0.313	0.006122	0.547
**I-31 days**	0.000	0.22	0.000	0.000	0.000	0.000	0.000	0.105
**I-6 months**	0.000	1.000	0.000	0.000	0.000	0.000	0.000	0.105
**II-4 hours**	1	1	1	1	1	1	0.021605	1
**II-14 days**	0.000	0.000	0.000	0.000	0.000	0.000	0.187036	0.047

**Legend**: IH = Intima hyperplasia, IIEL = irritation of internal elastic lamina, MFI = media fibromyocyte infiltration, N = necrosis, AH = adventitial hypercellularity, NL = neovascularization/lymphangiogenesis, ATEM. = amount of thromb-embolic material, T = volume of thrombotic material.

**Figure 3:**
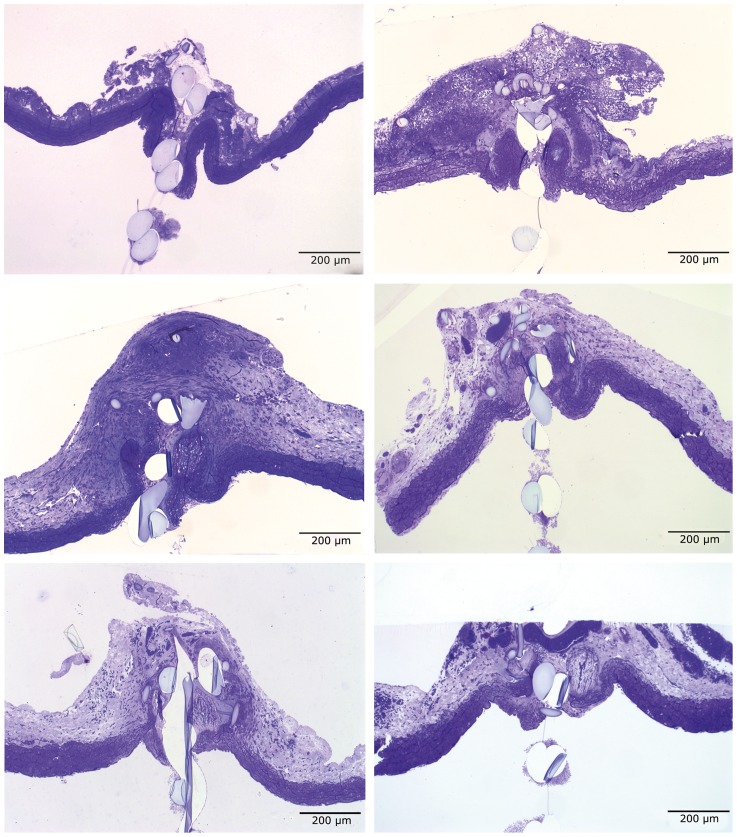
Typical histological changes and analyses of the specimen in groups I and II in *65x magnification*: *Upper left picture* 4 hours, *Upper right picture* 3 days, *middle left picture* 7 days, *middle right picture* 14 days, *lower left picture* 31 days and *lower right picture* 6 months postoperatively. (* = intimal proliferation, # = perivascular reaction, L = lumen, E = extravascular).

At postoperative day three, media necrosis was observed in a significant number compared to the control group (p<0.0001), but their histologic criteria were not found to be significantly different (**Figure** **3** **,** **Table 2** **)**.

Intimal hyperplasia (p = 0.005), fibromyoblast invasion of the media (p = 0.031), media necrosis (p<0.0001) and increased adventitial cellularity (p = 0.002) turned to be significantly detectable following a seven days interval (**Figure 3** **,** **Table 2** **)**. Two specimens showed neovascularization (p = 0.313). Intimal hyperplasia (p = 0.015) and adventitial hypercellularity were significantly increased (p = 0.002) compared to a 3 days interval (**Table 4** **)**.

**Table 4:** Statistical analysis of the histological changes using the Mann-Whitney-U Test and T-Test for T*.

Group	IH	IEI	MFI	N	AH	NL	T*
**3 vs. 7 days**	0.015	0.394	0.065	0.065	0.002	0.394	0.478
**3 vs. 14 days**	0.002	0.713	0.002	0.007	0.000	0.002	0.114
**3 vs. 31 days**	0.002	0.394	0.002	0.002	0.002	0.002	0.031
**3 vs. 6** **months**	0.002	0.699	0.002	0.002	0.002	0.002	0.031
**7 vs. 14 days**	0.093	0.181	0.022	0.562	0.492	0.016	0.118
**7 vs. 31 days**	0.065	0.093	0.002	0.394	0.394	0.015	0.031
**7 vs. 6** **months**	0.065	0.699	0.002	0.394	0.065	0.015	0.031
**14 vs. 31 days**	0.792	0.562	0.368	0.792	0.792	0.792	0.108
**14 days vs. 6** **months**	0.792	0.368	0.368	0.792	0.002	0.002	0.108
**31 days vs. 6** **months**	1.000	0.18	1.000	1.000	0.002	1.000	–

**Legend:** IH = Intima hyperplasia, IIEL = irritation of internal elastic lamina, MFI = media fibromyocyte infiltration, N = necrosis, AH = adventitial hypercellularity, NL = neovascularization/lymphangiogenesis, T = volume of thrombotic material.

All histologic parameters showed highly significant changes (each p<0.0001) 31 days postoperatively, except the irritation of the lamina elastica interna (p = 0.22).

**(** **Figure 3** **,** **Table 2** **)**. The fibromyoblast invasion of the media and media necrosis were significantly increased compared to the three and seven days sub-groups (each p = 0.002). The adventitia showed a significantly higher amount of cells compared to a three days interval (p = 0.002), but it was not significantly increased compared to a seven days interval (p = 0.394). The neovascularization and lymphangiogenesis in the adventitia occurred in a significant amount at 31 days compared to three (p = 0.002) and seven days, respectively (p = 0.015). Compared to a six months interval, however, differences were not significantly different (p = 0.97) (**Table 4** **)**. The histological findings showed no progression and remained localized six month postoperatively. The adventitial hypercellularity was significantly reduced (p = 0.002) with persistent neovascularization and lymphangiogenesis compared to the 31 days sub-group (p = 0.98) (**Figure 3** **)**. The adventitial hypercellularity showed no significant increase compared to a seven days interval (p = 0.065) but had a significant increase of neovascularization and lymphangiogenesis adjacent to the filter anastomosis (p = 0.015). Intimal hyperplasia was only significantly increased in comparison to the three days interval (p = 0.002); in comparison to the seven, 14 or 31 days there was no significant change detectable (p = 0.065, p = 0.792 and p = 0.98) (**Table 4** **)**.

### Group II

18 of 20 vessels were patent (90%), one vessel in each sub-group was occluded. 0.021605 mm^3^ (4 hours) and 0.187036 mm^3^ (14 days) of thrombembolic material was extruded in average, with a significantly increased amount of thrombembolic material after a 14 days interval compared to a four hours interval (p = 0.047) (**Table 3**).

No significant histological changes were seen four hours postoperatively. One filter was occluded due to traumatic proximal anastomosis, which became evident in the histologic work up (**Figure 3** **,** **4** **)**. No significant changes were detected in comparison between proximal non-filter anastomosis and distal filter anastomosis, including the irritation of lamina elastica interna (p = 0.796) (**Table 5** **)**.

**Figure 4:**
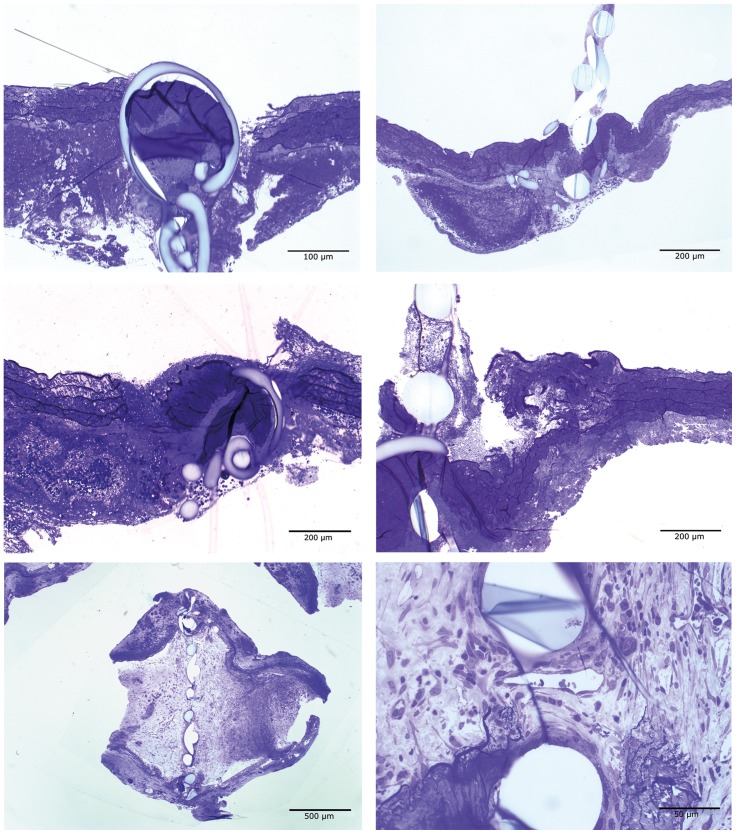
Typical histological changes of the occluded anastomoses: *Upper row* 4 hours postoperatively showing culpable, traumatic (t) proximal anastomosis (*left, 130x magnification*) with occluded filter anstomosis (*right, 65x magnification*); *middle row* 14 days postoperatively showing both traumatic proximal and distal anastomoses *(both 65x magnification)*; *lower row* 31 days postoperatively showing complete intravascular fibrotic (f) reorganisation with neovascularization (n) and lypmhangiogenesis (l) (*left 26x and right 260x magnification)*. (L = lumen, E = extravascular).

**Table 5:** Statistical analysis of the histological changes in the proximal and distal anastomoses of group II using the Mann-Whitney-U.

Group II	IH	IIEL	MFI	N	AH	NL
**4 hours proximal vs. distal**	1.000	0.796	1.000	1.000	1.000	1.000
**14 days proximal vs. distal**	0.005	0.529	0.043	1.000	1.000	0.011

**Abbreviations:** IH = Intima hyperplasia, IIEL = irritation of internal elastic lamina, MFI = media fibromyocyte infiltration, N = necrosis, AH = adventitial hypercellularity, NL = neovascularization/lymphangiogenesis, T = volume of thrombotic material.

A localized intimal proliferation (p = 0.005), media fibromyoblast invasion (p = 0.043), neovascularization and lymphangiogenesis (p = 0.002) adjacent to the filter (<500 µm) were seen in a higher extent in the distal filter anastomosis in comparison with proximal anastomosis 14 days postoperatively. The media necrosis 14 days postoperatively was completely demarcated compared to a three days interval (p = 0.007) and showed no significant progression after seven days (p = 0.562) (**Figure 3** **,** **Table 4** **)**. The filter of one rat was completely occluded three hours postoperatively due to inadequate proximal anastomosis (**Figure 4**).

## Discussion

Postoperative thrombosis and consecutive embolization is a life-threatening event and remains the main culprit behind failures in vascular surgery and free tissue transfer [8], [22], [23]. The results of prior work show that poor surgical handling during operative procedures in atherosclerotic vessels are significantly associated with thrombosis and secondary embolism that compromise the downstream microcirculatory system [24], [25], [26]. The use of additional techniques, such as fibrin based haemostatic agents or other tissue glues, might further increase this risk of thrombosis [27].

In the last decade, several “Embolization or Distal Protection Devices” (EPD/DPD) were introduced in vascular surgery and endovascular interventional radiology. These include proximal and distal balloons and filter-based or basket devices, which are mainly used in coronary, carotid and renal artery vascular interventions and are immediately removed after the procedure [15], [28]. Overall there seems to be a reduction in distal embolization when using such mentioned temporarily intravascular devices. A sensitive model to evaluate this life-threatening event of distal thrombembolism is missing. In addition, no permanent arterial filter has been introduced yet.

The described aorta-filter model is easily and individually applicable in microvessels (less than 3 mm diameter; 1.2–1.6 mm in diameter in the present study) with a reliable detection and prevention of thrombembolic events to the distal circulatory system. There is a wide range of application possibilities for this model, whether experimental or clinical. From the experimental point of view, short and long term thrombembolic risks of different procedures in vascular surgery or endovascular interventional radiology (new suturing techniques, thrombembolic risk of new vascular prosthesis, late onset embolism following vascular interventions using EPD/DPD, e.g.) can be critically and sensitively analysed. In clinical situations it might be of benefit of early detection of thrombosis following microvascular anastomosis and could reduce distal dislodgement and territorial infarction/necrosis of free tissue flaps. Secondary, revisions or salvage operations might be less traumatic and easier, if the filter was located in a good accessible vessel section.

In this study, the polyurethane filter anastomosis induced a localized foreign body reaction adjacent to the anastomosis with no disturbance of the physiological healing process. Overall, the healing process following vascular anastomosis and filter insertion was uneventful in postoperative histologic analysis and seemed to be finished after 14-day interval. Media necrosis started three days postoperatively and was accompanied by beginning intimal hyperplasia after a 7-day interval. Intimal hyperplasia and adventitial hypercellularity remained until a postoperative period of six months. The localized intimal hyperplasia following vascular anastomosis is reported to start after an interval of seven to ten days and may reach a thickness of approximately 2/3 of normal media and is caused among others by blood flow changes [29], [30]. Blood flow changes are known to have an important role on cell-cell interactions and gene expression in endothelial cells [31], [32]. The highly arranged adventitia was mainly made up of fibrocytes and fibroblasts that produced an increased amount of extracellular matrix. The strictly localized distribution of this feature reminds one of a protecting cuff surrounding the filter anastomosis and was seen following a 7–14 days interval (**Figure 3** **and** **4** **)**. Recent research support this finding, suggesting that adventitial fibroblasts are transformed into myofibroblasts and then migrate toward vessel lumen after proliferation to cause or induce intimal hyperplasia [33]. This might also explain the increased intimal hyperplasia in the filter anastomoses. In contrast to the work of others the increased intimal hyperplasia adjacent to the anastomosis was not associated with an increased number of thrombosed vessels [29].

There was no increase in postoperative thrombogenesis or in aneurysm formation. In one rat the aorta was completely occluded due to intravascular fibrosis and neovascularization/lymphangiogenesis (sub-group 31 days) (**Figure 4** **)**. Curiously, the rat did not show any clinical symptoms of thrombotic obliteration of the aortic bifurcation (“Leriche syndrome”), e.g. exhibition of pain reactions, intermittent claudication and global atrophy of the lower extremities [34]. This observation implies a slow progression of this vessel remodelling process. The blood supply to the distal body parts seemed to be maintained with collateral vessels.

One limitation of the presented study is the neglect of the endothelial cell layer in the postoperative analysis. The integrity of the endothelial cell layer and internal elastic lamina of the vessel wall plays crucial role in induction of thrombogenesis blood flow regulation. We neglected the special analysis of the endothelial cell layer and its integrity because the results would have been biased by the mechanical extraction of potential thromembolic material in the postoperative analysis. Furthermore, the potential re-rendothelialisation rate following filter insertion was not the objective of this study. But the analysis of the interruption of the internal elastic lamina and our high patency rate of 95.83% of the group I (**Table 2**) suggest how atraumatic this rather traumatic appearing procedure was. On the other hand, a traumatic handling and suturing will immediately result in vessel occlusion due to thrombogenesis and increased intimal hyperplasia, as is known from the literature [29], [35].

We strongly support the further use of this rat model. The histological changes did not influence the course of undisturbed vascular wound healing and appeared to prevent infarction of the distal vascular territory. Only a small amount of specialised equipment is needed and the whole surgical and anaesthetic procedure can be managed by the surgeon alone [20].

To the best of our knowledge this is the first study of arterial microvascular permanent filter device for the protection of distal vascular territories.

## Supporting Information

Figure S1 **ARRIVE Guideline.**
(PDF)
